# Predictors of All-Cause 30-Day Readmissions in Patients with Heart Failure at an Urban Safety Net Hospital: The Importance of Social Determinants of Health and Mental Health

**DOI:** 10.1016/j.ajmo.2023.100060

**Published:** 2023-09-30

**Authors:** Alexandra B. Steverson, Paul J. Marano, Caren Chen, Yifei Ma, Rachel J. Stern, Jean Feng, Efstathios D. Gennatas, James D. Marks, Matthew S. Durstenfeld, Jonathan D. Davis, Priscilla Y. Hsue, Lucas S. Zier

**Affiliations:** a Department of Medicine, University of California, San Francisco, Calif; b Division of Cardiology, Zuckerberg San Francisco General, San Francisco, Calif; c San Francisco Department of Public Health; d Ventura County Healthcare Agency, Ambulatory Care; e Department of Epidemiology and Biostatistics, University of California, San Francisco

**Keywords:** Heart failure, Readmission, Safety net hospital, Social determinants of health

## Abstract

**Introduction:**

Heart failure (HF) is a frequent cause of readmissions. Despite caring for underresourced patients and dependence on government funding, safety net hospitals frequently incur penalties for failing to meet pay-for-performance readmission metrics. Limited research exists on the causes of HF readmissions in safety net hospitals. Therefore, we sought to investigate predictors of 30-day all-cause readmission in HF patients in the safety net setting.

**Methods:**

We performed a retrospective chart review of patients admitted for HF from October 2018 to April 2019. We extracted data on demographics and medical comorbidities and performed patient-specific review of social determinants and mental health in 4 domains: race/ethnicity, housing status, substance use, and mental illness. Multivariable Poisson regression modeling was employed to evaluate associations with 30-day all-cause readmission.

**Results:**

The study population included 290 patients, among whom the mean age was 59 years and 71% (*n* = 207) were male; 42% (120) were Black/African American (AA), 22% (64) were Hispanic/Latino, and 96% (278) had public insurance; 28% (79) were not housed, 19% (56) had a diagnosis of mental illness, and active substance use was common. The 30-day readmission rate was 25.5% (*n* = 88). Factors that were associated with increased risk of readmission included self-identifying as Black/AA (relative risk 2.28, 95% confidence interval 1.00-5.20) or Hispanic/Latino (2.53, 1.07-6.00), experiencing homelessness (2.07, 1.21-3.56), living in a shelter (3.20, 1.27-8.02), or intravenous drug use (IVDU) (2.00, 1.08-3.70).

**Conclusion:**

Race/ethnicity, housing status, and substance use were associated with increased risk of 30-day all-cause readmission in HF patients in a safety net hospital. In contrast to prior studies, medical comorbidities were not associated with increased risk of readmission.

## Introduction

Heart failure is one of the most frequent causes of hospitalization in the United States, and patients with heart failure are frequently readmitted within 30 days. In 2010, the Centers for Medicare and Medicaid Services (CMS) established the Hospital Readmissions Reduction Program (HRRP) with the goal of reducing preventable hospitalizations by imposing financial penalties on hospitals with higher-than-expected 30-day readmission rates.^1^ Despite the significant need for governmental support, pay-for-performance readmission metrics disproportionately penalize safety net hospitals due to high readmission rates.^2^^,^^3^ Revised risk adjustment models to account for socioeconomic status based on the Medicare population's socioeconomic status resulted in a significant reduction in penalties for safety net hospitals after the 21st Century Cures Act, but disparities in penalties persist.^4^, ^5^

In patients with heart failure, medical comorbidities, biomarkers, and patient demographics may predict readmission.^6^^,^^7^ Additionally, socioeconomic status, which is often represented broadly by income level, educational attainment, employment status, and race/ethnicity, is associated with heart failure readmission.^8^^,^^9^ Social determinants of health (SDOH), defined as the economic, social, environmental, and psychosocial factors that influence health,^10^ may in part explain the high readmission penalties in safety net settings. However, patient-specific social determinant and mental health variables are often not included in studies of readmissions because they are not available. Instead, proxies such as ZIP code–based socioeconomic data are often relied upon to represent SDOH variables.^11^^,^^12^

Therefore, the objective of this study was to evaluate the effect of previously identified risk factors for readmission, such as demographics and medical comorbidities, and patient-specific social determinants and mental health variables  in the domains of race/ethnicity, housing status, substance use, and mental illness on the risk of 30-day all-cause readmissions in a heart failure population at an urban safety net hospital.

## Methods

We retrospectively identified all patients discharged with a diagnosis of heart failure from an urban safety net hospital in San Francisco from October 2018 to April 2019. Patients were initially identified by International Classification of Diseases 10th Revision (ICD-10) primary or secondary discharge diagnosis code for HF exacerbation. Two physicians then independently adjudicated the medical record of each patient to confirm that identified patients were treated for a heart failure exacerbation during their admission. Treatment for heart failure was defined as receiving at least 1 guideline-directed medication for an acute heart failure exacerbation during their admission. We excluded patients if they were less than 21 years of age or died during the index hospital admission. The institutional review board at the University of California San Francisco approved this study.

Demographic information including age, self-identified gender, primary language, and insurance status was abstracted from administrative data. Medical comorbidities were obtained by ICD-10 diagnosis codes for the index hospitalization and confirmed by review of the medical record. Medical comorbidities included atrial fibrillation, coronary artery disease (including prior MI), CKD, diabetes mellitus, hyperlipidemia, hypertension, stroke, COPD, and obesity and were selected in concordance with previous research evaluating predictors of HF readmission.^6^^,^^13^^,^^14^

Heart failure classification was determined by 2-physician review (AS, PM) of the medical record including the most recent echocardiogram. Patients were defined as heart failure with reduced ejection fraction (HFrEF) if their left ventricular ejection fraction (LVEF) was less than or equal to 40%. Heart failure with preserved ejection fraction (HFpEF) was defined as an LVEF of greater than 40%. Patients were defined as heart failure with improved ejection fraction (HF improved EF) if an echocardiogram prior to the index admission demonstrated LVEF less than or equal to 40% with a subsequent echocardiogram showing an LVEF >40%. These classifications were selected prior to release of the most recent American Heart Association / American College of Cardiology heart failure guidelines.

Using ICD-10 codes for diagnostic identification of SDOH and mental illness can be inaccurate, particularly when used to determine housing status and substance use history.^11^^,^^12^ Therefore, we directly abstracted variables using the following protocol:

1) Race/ethnicity was self-reported by patients upon admission and this data was abstracted directly from the medical record.
2) Housing status was identified upon 2-physician review of the medical record. Patients were defined as housed if they were discharged to a residential property with a discrete address identified as their own. Patients were defined as marginally housed if they were discharged to a discrete residential property that was identified as temporary housing. Patients were defined as sheltered if they were discharged to a homeless shelter or recuperative care. Finally, patients were identified as experiencing homelessness if they were discharged without an identified residential property, shelter, or recuperative care.^15^
3) Substance use was identified by detailed 2-physician chart review. We defined substance use as a documented history of alcohol, opioid, methamphetamine, and/or cocaine use in the past medical history, social history, and/or a urine or serum toxicology screen identifying the presence of one or more of these substances at the index admission. Intravenous drug use (IVDU) was identified from review of the medical record.
4) Mental illness was identified by review of ICD-10 code for the index hospitalization and then confirmed by 2-physician chart review. Diagnoses included were recognized by the Diagnostic and Statistical Manual of Mental Disorders, Fifth Edition as mental illnesses. Given the prevalence of substance use disorders in this cohort and the importance of evaluating both mental illness and substance use as predictors of readmission in the model, these diagnostic categories were characterized separately (see #3).

When there was discordance in physician abstraction of these variables, the charts were reviewed independently by multiple authors (AS, PM, LZ) and consensus reached.

We defined a 30-day all-cause readmission event as any admission within 30 days of discharge from the index hospitalization excluding elective surgical procedures. Thirty-day all-cause readmission events occurring at outside hospitals were captured if admission data was linked from outside electronic medical record (EMR). This cohort included only patients requiring hospitalization for HF, which is by definition New York Heart Association (NYHA) class IV HF. Severity of cardiomyopathy is also represented by LVEF; however, this does not entirely capture the severity of HF. We were interested in the effect of the variables on readmission and hypothesized that severity of disease is a mediator not a confounder in this analysis.

We used chi-square or Fisher exact tests to compare categorical variables. Wilcoxon rank sum test was used to compare continuous variables as normality was not met. We counted the number of readmission events at the patient level. The distribution of readmission was right skewed and the variance was about the same as the mean which suggests there was no overdispersion in the data. Therefore, we fit a Poisson regression model to evaluate the impact of risk factors on 30-day all-cause readmission. The exponentiated regression coefficients represent the relative risk (RR) of readmission. We verified the model assumption of equidispersion (ie, conditional mean = variance) by goodness-of-fit chi-square test and, therefore, confirmed the adequacy of the Poisson regression model. We applied the model to the entire cohort and performed a sensitivity analysis on the group of HFrEF patients. We chose variables for multivariable adjustment that were significant on univariable analysis and had been shown to be previously associated with readmission. All analyses were performed using SAS 9.4 (SAS Institute, Cary, NC). The first and senior author had full access to all the data in the study and take responsibility for its integrity and for the data analysis.

## Results

We identified 290 patients who were hospitalized for an HF exacerbation during the study period; 88 patients experienced a 30-day readmission (*n* = 88, 25.5%), and of these, 13 patients experienced 2 or more readmissions. The median length of stay was 4 days.

Clinical characteristics are outlined in Table 1. Mean age was 59 (SD 13) and 71% (*n* = 207) were male. In total, 42% of patients (*n* = 120) identified as Black/AA and 22% (*n* = 64) identified as Hispanic/Latino. All patients were insured; 96% (*n* = 278) had public insurance, with 40% (*n* = 116) enrolled in Medicare, 22% (*n* = 63) enrolled in Medicaid, and 34% (*n* = 99) enrolled in county subsidized public health insurance (San Francisco Health Network); 28% (*n* = 81) were experiencing homelessness, living in a shelter, or marginally housed; 27% (*n* = 78) used methamphetamines, 35% (*n* = 101) used cocaine, 16% (*n* = 45) used opioids, and 33% (*n* = 96) used alcohol; and 10% (*n* = 29) of patients used intravenous drugs.

**Table 1:** Baseline Characteristics of Heart Failure Patients.

Characteristic	
(*n* = 290)	
Self-reported race/ethnicity	
White	48 (16.6%)
Black/AA	120 (41.5%)
Hispanic/Latino	64 (22.1%)
Asian	45 (15.6%)
Other	12 (4.2%)
Self-identified gender	
Male	207 (71.4%)
Female	83 (28.6%)
Age (years, SD)	59 (13)
Language	
English	238 (82.6%)
Spanish	27 (9.4%)
Cantonese	11 (3.8%)
Other	12 (4.2%)
HF classification	
HFpEF	71 (24.5%)
HFrEF	211 (72.8%)
HF improved EF	8 (2.8%)
Ejection fraction (interquartile)	30 (20, 45)
Ejection fraction <40%	211 (72.8%)
Atrial fibrillation	101 (34.8%)
CAD	136 (46.9%)
CKD	149 (51.4%)
Diabetes mellitus	105 (36.2%)
Hyperlipidemia	66 (22.8%)
Hypertension	217 (74.8%)
Obesity	40 (13.8%)
Stroke	2 (0.7%)
COPD	98 (33.8%)
Mental illness	56 (19.3%)
Tobacco use	185 (63.8%)
Alcohol use	96 (33.1%)
Opioid use	45 (15.5%)
Cocaine use	101 (34.8%)
Methamphetamine use	78 (26.9%)
IVDU	29 (10%)
GDMT use for those with LVEF <40% (*n* = 211)	
Beta-blocker prescribed	170 (80.6%)
ACE inhibitor or ARB prescribed	160 (76.2%)
MRA prescribed	76 (36.0%)
Housing status	
Homeless	48 (16.6%)
Housed	209 (72.1%)
Marginal	24 (8.3%)
Shelter	9 (3.1%)
Insurance status	
Medicare	116 (40%)
Medi-Cal	63 (21.7%)
SF Health Network	99 (34.1%)
Other	12 (4.1%)
Length of stay, day, median (interquartile)	4 (2, 7)
All-cause 30-day readmission rate	74 (25.5%)
Patients with 1 readmission	61 (21%)
Patients with 2 readmissions	12 (4.1%)
Patients with 3 readmissions	1 (0.3%)

The mean EF was 30% (interquartile range 20, 45), and 73% of patients (*n* = 211) had an EF <40%. Medical comorbidities included atrial fibrillation (*n* = 101, 35%), coronary artery disease (*n* = 136, 47%), hypertension (*n* = 217, 75%), diabetes mellitus (*n* = 105, 36%), hyperlipidemia (*n* = 66, 23%), obesity (*n* = 40, 14%), CKD (*n* = 149, 51%), stroke (*n* = 2, 0.7%), and COPD (*n* = 98, 34%). Also, 19% (*n* = 56) had a diagnosis of mental illness.

Use of GDMT in those with an LVEF of <40% was common. On discharge from the hospital, 81% of patients (*n* = 170) were prescribed a beta-blocker, 76% (*n* = 160) were prescribed an ACE inhibitor or ARB, and 36% (*n* = 76) were prescribed an MRA. It was standard practice to provide 30 days of medications at discharge after HF exacerbation to all patients receiving medication.

On univariable analysis (Table 2) SDOH variables and substance use disorders were associated with increased risk of 30-day readmission. Race/ethnicity (*P* = .01), housing status (*P* = .004), insurance type (*P* = .05), and substance use (including methamphetamine use [*P* = .03], alcohol use [*P* = .02], and IVDU [*P* < .001]) were associated with all-cause 30-day readmission. No medical comorbidities were associated with all-cause 30-day readmission, including all HF types (HFrEF, HFpEF, and HF with improved EF).

**Table 2:** Univariable Analysis of Variables Associated with All-Cause 30-Day Readmission in Heart Failure Patients.

	No readmission (*n* = 216)	Readmission (*n* = 74)	*P* value
Age, mean	61	60	.56
Self-reported race/ethnicity			.01
White	41 (19.5%)	7 (9.5%)	
Black/AA	84 (38.9%)	36 (48.6%)	
Hispanic/Latino	42 (19.4%)	22 (29.7%)	
Asian	41 (19.0%)	4 (5.4%)	
Other	7 (3.2%)	5 (6.8%)	
Self-identified gender			.72
Male	153 (70.8%)	54 (73.0%)	
Female	63 (29.2%)	20 (27.0%)	
Language			.54
English	175 (81.0%)	63 (85.1%)	
Spanish	19 (8.8%)	8 (10.8%)	
Cantonese	10 (4.6%)	1 (1.4%)	
Other	10 (4.6%)	2 (2.7%)	
Insurance status			.05
Medicare	89 (41.2%)	27 (36.5%)	
Medicaid	49 (22.7%)	14 (18.9%)	
SFHN	66 (30.6%)	33 (44.6%)	
Other	12 (5.6%)	0	
Housing status			.004
Homeless	28 (13.0%)	20 (27.0%)	
Housed	166 (76.9%)	43 (58.1%)	
Marginal	18 (8.3%)	6 (8.1%)	
Shelter	4 (1.9%)	5 (6.8%)	
Mental illness	40 (18.5%)	16 (21.6%)	.56
Methamphetamine use	51 (23.6%)	27 (36.5%)	.03
Cocaine use	69 (31.9%)	32 (43.2%)	.08
Opioid use	31 (14.4%)	14 (18.9%)	.35
Tobacco use	134 (62.0%)	51 (68.9%)	.29
Alcohol use	63 (29.2%)	33 (44.6%)	.02
IVDU	13 (6.0%)	16 (21.6%)	<.0001
Medical comorbidities			
Atrial fibrillation	77 (35.6%)	24 (32.4%)	.62
CAD	103 (47.7%)	33 (44.6%)	.65
CKD	110 (50.9%)	39 (52.7%)	.79
Diabetes mellitus	74 (34.3%)	31 (41.9%)	.24
Hyperlipidemia	49 (22.7%)	17 (23.0%)	.96
Hypertension	161 (74.5%)	56 (75.7%)	.85
Obesity	34 (15.7%)	6 (8.1%)	.10
Stroke	1 (0.5%)	1 (1.4%)	.42
COPD	69 (31.9%)	29 (39.2%)	.26
HF type			.43
HFpEF	57 (26.4%)	14 (18.9%)	
HFrEF	153 (70.8%)	58 (78.4%)	
Improved EF	6 (2.6%)	2 (2.7%)	
Cardiology follow-up within 7 days	96 (44.4%)	33 (44.6%)	.98
PCP follow-up within 7 days	126 (58.3%)	50 (67.6%)	.16
Dry weight documented at discharge	120 (55.6%)	30 (40.5%)	.06

On multivariable analysis (Table 3), similar trends persisted. Identifying as Hispanic/Latino (RR 2.5, 95% confidence interval [CI] 1.1-5.9, *P* = .04) or Black/AA (2.3,1.0-5.2, *P* = .05) was associated with increased risk of 30-day readmission. There was a significant association for patients experiencing unstable housing, either shelter use (3.2,1.3-7.9, *P* = .01) or experiencing homelessness (2.1, 1.2-3.5, *P* = .01). IVDU was significantly associated with increased risk of readmission (2.3, 1.4-4.0, *P* = .002). In the multivariable model, there was no association of medical comorbidities with an increased risk of 30-day all-cause readmission, including all HF types (HFrEF, HFpEF, and HF with improved EF).

**Table 3:** Multivariable Poisson Regression Model of Variables Associated with All-Cause 30-Day Readmissions in Heart Failure Patients.

Characteristic	Relative risk (95% CI)	*P* value
Age (years)	1.00 (0.98-1.02)	.87
Self-identified gender		
Male	1.0	
Female	1.24 (0.72-2.11)	.44
CAD	0.82 (0.52-1.29)	.39
Hypertension	1.15 (0.67-1.95)	.62
Diabetes	1.30 (0.82-2.05)	.26
CKD	1.14 (0.73-1.79)	.57
COPD	0.86 (0.53-1.39)	.54
HF type		
HFrEF	1.0	
HFpEF	0.92 (0.52-1.62)	.76
Improved EF	0.75 (0.17-3.30)	.70
Self-reported Race/ethnicity		
White	1.0	
Black/AA	2.27 (1.00-5.16)	.05
Hispanic/Latino	2.50 (1.05-5.92)	.04
Asian	0.66 (0.19-2.31)	.51
Other	3.03 (1.00-9.16)	.05
Mental illness	1.15 (0.66-2.01)	.61
Housing status		
Housed	1.0	
Marginally housed	1.01 (0.43-2.35)	.98
Shelter	3.16 (1.26-7.93)	.01
Homeless	2.06 (1.19-3.54)	.01
Intravenous Drug use	2.34 (1.36-4.01)	.002

On sensitivity analysis (Table 4), similar trends persisted in HFrEF patients. Identifying as Hispanic/Latino (2.97, 1.0-8.9, *P* = .05) was associated with increased risk of readmission. The effect of identifying as Black/AA is similar but the significance level was attenuated (2.61, 0.91-7.50, *P* = .08). There was a significant association for patients experiencing unstable housing, either shelter use (3.6, 1.4-9.3, *P* = .01) or experiencing homelessness (2.3, 1.3-4.3, *P* = .01). IVDU was significantly associated with increased risk of readmission (1.9, 1.0-3.5, *P* = .05).

**Table 4:** Sensitivity Analysis of Multivariable Poisson Regression Model for All-Cause 30-Day Readmission Among HFrEF Patients

Characteristic	Relative risk (95% CI)	*P* value
Age (years)	1.00 (0.98-1.03)	.83
Self-identified gender		
Male	1.0	
Female	1.00 (0.50-2.02)	.99
Diabetes mellitus	1.17 (0.69-1.98)	.57
Hypertension	1.37 (0.76-2.49)	.29
COPD	0.88 (0.50-1.53)	.65
CAD	0.72 (0.43-1.21)	.22
CKD	1.26 (0.75-2.11)	.38
Mental illness	1.27 (0.67-2.44)	.46
Self-reported race		
White	1.0	
Black/AA	2.61 (0.91-7.50)	.08
Hispanic/Latino	2.97 (1.00-8.87)	.05
Asian	0.88 (0.19-4.10)	.88
Other	4.54 (1.26-16.26)	.02
Housing status		
Housed	1.0	
Marginal housing	0.98 (0.36-2.65)	.97
Shelter	3.60 (1.39-9.33)	.01
Homeless	2.33 (1.27-4.26)	.01
Intravenous drug use	1.89 (1.01-3.51)	.05

## Discussion

We found that among individuals hospitalized for heart failure at a safety net hospital, experiencing homelessness, self-identification as Black/AA or Hispanic/Latino, and substance use were associated with increased risk of all-cause 30-day readmission. In contrast to prior studies, we did not identify medical comorbidities associated with increased risk of readmission in this population.

There are several reasons why  social determinants and mental illness such as substance use disorders  may be associated with increased risk of readmission. We found that race/ethnicity, specifically identifying as Black/AA or Hispanic/Latino, was significantly associated with increase in risk of 30-day all-cause readmission, which is similar to prior studies.^16^^,^^17^ This finding raises concern regarding institutionalized and structural racism in the health care system. Despite a wealth of data acknowledging disparities in prevalence of CVD, readmission rates, and cardiovascular mortality in Black/AA patients, there is a lack of research on the effects of racism in these contexts and the mechanisms by which it relates to these disparities, which must be addressed.^10^^,^^18^

There has also been much focus on experiencing homelessness as a driver of disease. Individuals who experience homelessness carry a high burden of comorbid medical illness and experience accelerated aging.^19^ Increasing disease burden has been associated with elevated readmission rates.^20^ Despite evidence of significant medical comorbidities, individuals experiencing homelessness are less likely to obtain longitudinal ambulatory care and are more likely to use acute care services and emergency departments.^21^ Heart failure is a disease in which coordinated, longitudinal care is particularly important and the inability of persons experiencing homelessness to access this key component of heart failure therapy may, in part, explain their increased risk of readmission. Basic tenets of outpatient heart failure therapy such as medication adherence and daily weights are made more complex without stable housing. Needs such as securing adequate food and shelter may compete with activities of daily heart failure care. Finally, as a population, persons experiencing homelessness have a 5-fold increase in baseline hospitalization rate compared to housed persons.^22^ Thus, hospitals that care for a significant number of unstably housed heart failure patients may experience elevated readmission risk.

Potential mechanisms contributing to increased all-cause 30-day readmission risk in heart failure patients who actively use substances may be no less substantial. Prior research has shown that patients who are actively using substances exhibit particularly poor adherence to medical therapy.^23^ In addition, these individuals may have difficulty undergoing treatment for medical comorbidities, such as heart failure, until their substance use disorder has been addressed.^24^ Substance use disorders have been independently associated with increased emergency room visits and utilization of acute inpatient services in patients with heart failure.^25^ Thus, patients with both substance use disorders and heart failure may experience elevated readmission risk.

Finally, it is important to recognize that certain social determinants  have been shown to be associated with higher rates of mental illness such as substance use disorders.^26^ Specifically, in heart failure, multimorbidity is common, including concurrent substance use and mental illness.^27^ The effect of these variables may be additive, making it particularly difficult for patients to engage in effective treatment for heart failure.

Safety net providers are unlikely to be surprised by the results of this study. Heart failure in combination with 1 or more adverse SDOH factors often shifts the treatment paradigm toward addressing social needs first or at a minimum in concert with heart failure. Strategies to address experiencing homelessness with, for example, recuperative care or substance use with inpatient addiction teams have shown some promise in reducing readmission rates.^28^^,^^29^ Addressing homelessness and substance use are beyond the expertise of inpatient cardiologists and may benefit from multidisciplinary teams and additional resources. Efforts to address SDOH and substance use disorders are critically important to reduce readmission rates and should be the primary focus of performance improvement efforts in the safety net setting.

While these findings should be confirmed in larger, multicenter studies, the implications of our findings on risk adjustment may be important. Researchers who study the effect of the HRRP and pay-for-performance readmission programs have begun to advocate for changes specifically involving penalties for safety net hospitals.^30^ Our findings support the suggestion to evolve risk adjustment models to better capture patient-specific social determinant and mental health variables rather than broader indirect measures of socioeconomic status. Additionally, the challenges faced by safety net hospitals in reducing 30-day readmissions require more, not less, resources to address homelessness and substance use.

This study was performed in a single safety net hospital with a relatively small study population. The analysis was retrospective and thus limits causal inference. The size of this study allowed us to perform a detailed investigation of demographics, medical comorbidities, social determinants, and mental health variables ; however, these results should be confirmed in other safety net settings and larger patient populations. Universal insurance access in San Francisco County may limit external generalizability. Medical comorbidities and mental illness diagnoses were identified using ICD-10 diagnosis codes which have demonstrated underestimation of disease in prior studies. We attempted to mitigate this risk by concurrently reviewing the medical record for each patient, but underestimation may have persisted. We based our analysis on patient-level factors which were abstracted from the EMR directly. Variables such as NYHA class or AHA/ACC stage, education level, income, and employment status were not available at a high enough rate to be included in the analysis. It is possible that readmission events occurred at outside hospitals that we were unable to capture due to limitations in linked outside EMR data. Finally, we elected to include race/ethnicity as a variable in our study to make comparisons to prior research and adjust for unmeasured confounding, but it should be noted that the inclusion of race/ethnicity as a variable in risk prediction models inherently perpetuates structural racism and may limit investigation into the underlying social determinants or unmeasured factors that may be associated with a specific outcome of interest.

## Conclusions

In an urban safety net hospital, caring for an underresourced heart failure population, we found that patient-reported race/ethnicity, housing status, and substance use were associated with an increased risk of all-cause 30-day readmission. In contrast to prior studies, we did not identify specific medical comorbidities that were associated with increased risk of readmission in this population. These findings help shed light on the underlying association of SDOH and mental health on readmission rates in the safety net setting and offer potential targets for policy and performance improvement initiatives aimed at reducing readmission rates.

## Sources of Funding

Internal grant from the San Francisco General Hospital Foundation.

## Disclosures

None.

## Declaration of Competing Interest

The authors declare that they have no known competing financial interests or personal relationships that could have appeared to influence the work reported in this paper.
